# Supplementary material for: Strong Purifying Selection at Synonymous Sites in D. melanogaster
Source: PLoS Genet. 2013 May 30;9(5):e1003527. doi: 10.1371/journal.pgen.1003527 (PMC3667748; doi:10.1371/journal.pgen.1003527)
Supplement: Dataset S1 — Genes enriched for high constraint. Table of the top 812 genes enriched for high constraint at 4D sites. (DOC) [file pgen.1003527.s001.doc]

**Dataset S1**. **Genes enriched for high constraint.**

| **FLYBASE GENE ID** | FBgn0021800 | FBgn0024273 | FBgn0033934 | FBgn0030847 | FBgn0003502 |
| --- | --- | --- | --- | --- | --- |
| FBgn0037245 | FBgn0022764 | FBgn0029936 | FBgn0046258 | FBgn0010422 | FBgn0017579 |
| FBgn0033029 | FBgn0025712 | FBgn0053995 | FBgn0040571 | FBgn0051637 | FBgn0035283 |
| FBgn0037739 | FBgn0003279 | FBgn0003659 | FBgn0037777 | FBgn0027052 | FBgn0001297 |
| FBgn0028373 | FBgn0032021 | FBgn0033108 | FBgn0033558 | FBgn0000618 | FBgn0039427 |
| FBgn0027550 | FBgn0040636 | FBgn0030870 | FBgn0040318 | FBgn0027565 | FBgn0036844 |
| FBgn0031632 | FBgn0029974 | FBgn0038100 | FBgn0036039 | FBgn0003360 | FBgn0036317 |
| FBgn0014163 | FBgn0016131 | FBgn0004654 | FBgn0000289 | FBgn0003205 | FBgn0020245 |
| FBgn0016061 | FBgn0037313 | FBgn0034636 | FBgn0039237 | FBgn0030260 | FBgn0027546 |
| FBgn0032162 | FBgn0042199 | FBgn0020647 | FBgn0040715 | FBgn0046687 | FBgn0035630 |
| FBgn0031637 | FBgn0039200 | FBgn0004862 | FBgn0035753 | FBgn0031816 | FBgn0026376 |
| FBgn0038763 | FBgn0035866 | FBgn0032297 | FBgn0013433 | FBgn0034421 | FBgn0037647 |
| FBgn0010704 | FBgn0034454 | FBgn0038063 | FBgn0050185 | FBgn0039969 | FBgn0000038 |
| FBgn0017581 | FBgn0052137 | FBgn0004118 | FBgn0008636 | FBgn0037697 | FBgn0026533 |
| FBgn0031126 | FBgn0025806 | FBgn0027600 | FBgn0001134 | FBgn0024232 | FBgn0000581 |
| FBgn0002789 | FBgn0031322 | FBgn0051365 | FBgn0030294 | FBgn0015754 | FBgn0020617 |
| FBgn0011676 | FBgn0031835 | FBgn0038092 | FBgn0017550 | FBgn0024187 | FBgn0039130 |
| FBgn0035873 | FBgn0040823 | FBgn0023423 | FBgn0015919 | FBgn0036044 | FBgn0036428 |
| FBgn0038126 | FBgn0033639 | FBgn0032181 | FBgn0035329 | FBgn0000308 | FBgn0047135 |
| FBgn0014026 | FBgn0026263 | FBgn0037521 | FBgn0036921 | FBgn0033480 | FBgn0039561 |
| FBgn0010894 | FBgn0000183 | FBgn0030361 | FBgn0025574 | FBgn0027657 | FBgn0015521 |
| FBgn0038805 | FBgn0039636 | FBgn0011227 | FBgn0004583 | FBgn0022985 | FBgn0065032 |
| FBgn0035500 | FBgn0035987 | FBgn0010762 | FBgn0035578 | FBgn0027497 | FBgn0036728 |
| FBgn0033174 | FBgn0082582 | FBgn0034861 | FBgn0034460 | FBgn0038619 | FBgn0051221 |
| FBgn0035236 | FBgn0034788 | FBgn0013954 | FBgn0034538 | FBgn0043012 | FBgn0031940 |
| FBgn0003386 | FBgn0014879 | FBgn0038098 | FBgn0035475 | FBgn0029687 | FBgn0022085 |
| FBgn0042112 | FBgn0004636 | FBgn0035400 | FBgn0004873 | FBgn0020238 | FBgn0035495 |
| FBgn0014020 | FBgn0038581 | FBgn0043364 | FBgn0015550 | FBgn0051005 | FBgn0028961 |
| FBgn0033907 | FBgn0037336 | FBgn0039159 | FBgn0035033 | FBgn0015778 | FBgn0033122 |
| FBgn0038515 | FBgn0023179 | FBgn0052485 | FBgn0000150 | FBgn0037814 | FBgn0035528 |
| FBgn0035981 | FBgn0039380 | FBgn0051216 | FBgn0017572 | FBgn0000339 | FBgn0024244 |
| FBgn0036337 | FBgn0000273 | FBgn0032588 | FBgn0026077 | FBgn0003941 | FBgn0042185 |
| FBgn0061361 | FBgn0035936 | FBgn0004572 | FBgn0034886 | FBgn0038589 | FBgn0003527 |
| FBgn0037664 | FBgn0002626 | FBgn0031955 | FBgn0028646 | FBgn0033925 | FBgn0039489 |
| FBgn0050373 | FBgn0035542 | FBgn0010382 | FBgn0035807 | FBgn0022382 | FBgn0033624 |
| FBgn0034091 | FBgn0034245 | FBgn0004363 | FBgn0027779 | FBgn0036364 | FBgn0003312 |
| FBgn0028331 | FBgn0051635 | FBgn0052260 | FBgn0037328 | FBgn0039581 | FBgn0004369 |
| FBgn0027356 | FBgn0034371 | FBgn0031627 | FBgn0001098 | FBgn0003721 | FBgn0036992 |
| FBgn0033985 | FBgn0030893 | FBgn0053100 | FBgn0039816 | FBgn0001319 | FBgn0038294 |
| FBgn0020300 | FBgn0027950 | FBgn0031310 | FBgn0000097 | FBgn0030357 | FBgn0000529 |
| FBgn0050158 | FBgn0042135 | FBgn0010226 | FBgn0051632 | FBgn0000181 | FBgn0010909 |
| FBgn0001078 | FBgn0022343 | FBgn0028871 | FBgn0033236 | FBgn0035335 | FBgn0004552 |
| FBgn0004177 | FBgn0010415 | FBgn0051121 | FBgn0034030 | FBgn0034399 | FBgn0013751 |
| FBgn0032504 | FBgn0011455 | FBgn0003326 | FBgn0020303 | FBgn0004907 | FBgn0036003 |
| FBgn0035281 | FBgn0011481 | FBgn0041087 | FBgn0031589 | FBgn0033309 | FBgn0042138 |
| FBgn0039617 | FBgn0037680 | FBgn0025286 | FBgn0021967 | FBgn0002945 | FBgn0052177 |
| FBgn0036556 | FBgn0004795 | FBgn0027589 | FBgn0030101 | FBgn0029715 | FBgn0031106 |
| FBgn0002973 | FBgn0027951 | FBgn0035085 | FBgn0001942 | FBgn0033544 | FBgn0039223 |
| FBgn0050295 | FBgn0031413 | FBgn0039748 | FBgn0005671 | FBgn0027527 | FBgn0034345 |
| FBgn0037728 | FBgn0000250 | FBgn0010877 | FBgn0014859 | FBgn0032465 | FBgn0035688 |
| FBgn0030850 | FBgn0034802 | FBgn0011211 | FBgn0010397 | FBgn0013973 | FBgn0037614 |
| FBgn0004921 | FBgn0023212 | FBgn0029944 | FBgn0015790 | FBgn0005533 | FBgn0051481 |
| FBgn0051146 | FBgn0038947 | FBgn0002567 | FBgn0015371 | FBgn0005640 | FBgn0011281 |
| FBgn0024734 | FBgn0032629 | FBgn0010269 | FBgn0000577 | FBgn0038654 | FBgn0024963 |
| FBgn0052405 | FBgn0035043 | FBgn0004611 | FBgn0003429 | FBgn0004179 | FBgn0027619 |
| FBgn0004618 | FBgn0004396 | FBgn0031661 | FBgn0037847 | FBgn0033782 | FBgn0010228 |
| FBgn0028622 | FBgn0028406 | FBgn0021895 | FBgn0052240 | FBgn0029708 | FBgn0043458 |
| FBgn0036111 | FBgn0030834 | FBgn0002940 | FBgn0030328 | FBgn0045038 | FBgn0026379 |
| FBgn0010348 | FBgn0032447 | FBgn0002283 | FBgn0034730 | FBgn0038267 | FBgn0010399 |
| FBgn0035844 | FBgn0005777 | FBgn0004919 | FBgn0011217 | FBgn0036851 | FBgn0051176 |
| FBgn0004101 | FBgn0039790 | FBgn0040297 | FBgn0038981 | FBgn0040551 | FBgn0027605 |
| FBgn0034021 | FBgn0028474 | FBgn0036223 | FBgn0011739 | FBgn0035367 | FBgn0010411 |
| FBgn0038683 | FBgn0030257 | FBgn0004889 | FBgn0033486 | FBgn0033452 | FBgn0051660 |
| FBgn0026250 | FBgn0039381 | FBgn0032101 | FBgn0039735 | FBgn0052428 | FBgn0005677 |
| FBgn0030456 | FBgn0032901 | FBgn0035853 | FBgn0012344 | FBgn0025455 | FBgn0034755 |
| FBgn0015324 | FBgn0038659 | FBgn0004892 | FBgn0010078 | FBgn0011278 | FBgn0028516 |
| FBgn0037448 | FBgn0037891 | FBgn0038855 | FBgn0038946 | FBgn0028582 | FBgn0002577 |
| FBgn0035601 | FBgn0011766 | FBgn0001139 | FBgn0000039 | FBgn0020910 | FBgn0034084 |
| FBgn0036436 | FBgn0033784 | FBgn0044323 | FBgn0037539 | FBgn0034408 | FBgn0011589 |
| FBgn0003275 | FBgn0038140 | FBgn0029067 | FBgn0039116 | FBgn0040384 | FBgn0004387 |
| FBgn0035982 | FBgn0021979 | FBgn0015299 | FBgn0040286 | FBgn0000099 | FBgn0038839 |
| FBgn0013726 | FBgn0033209 | FBgn0000108 | FBgn0029128 | FBgn0003411 | FBgn0029975 |
| FBgn0035323 | FBgn0030791 | FBgn0028342 | FBgn0029896 | FBgn0035558 | FBgn0050419 |
| FBgn0000157 | FBgn0031799 | FBgn0032305 | FBgn0030089 | FBgn0038494 | FBgn0024753 |
| FBgn0037747 | FBgn0016700 | FBgn0029911 | FBgn0000591 | FBgn0035533 | FBgn0011648 |
| FBgn0028789 | FBgn0036781 | FBgn0019662 | FBgn0062413 | FBgn0037358 | FBgn0003425 |
| FBgn0030873 | FBgn0000061 | FBgn0033668 | FBgn0050446 | FBgn0035792 | FBgn0050147 |
| FBgn0039225 | FBgn0001235 | FBgn0031866 | FBgn0030234 | FBgn0015591 | FBgn0011656 |
| FBgn0036685 | FBgn0033736 | FBgn0041184 | FBgn0013325 | FBgn0016726 | FBgn0034138 |
| FBgn0031359 | FBgn0051191 | FBgn0025633 | FBgn0030183 | FBgn0063649 | FBgn0027607 |
| FBgn0000635 | FBgn0039266 | FBgn0053505 | FBgn0016797 | FBgn0014001 | FBgn0039260 |
| FBgn0016691 | FBgn0034501 | FBgn0036126 | FBgn0036974 | FBgn0037770 | FBgn0011581 |
| FBgn0038964 | FBgn0024944 | FBgn0038877 | FBgn0032223 | FBgn0000564 | FBgn0001122 |
| FBgn0040512 | FBgn0052672 | FBgn0000115 | FBgn0015904 | FBgn0003896 | FBgn0034763 |
| FBgn0052843 | FBgn0001085 | FBgn0029768 | FBgn0051772 | FBgn0052057 | FBgn0031945 |
| FBgn0038065 | FBgn0035505 | FBgn0032499 | FBgn0083228 | FBgn0011224 | FBgn0010280 |
| FBgn0031692 | FBgn0051641 | FBgn0015799 | FBgn0038787 | FBgn0002593 | FBgn0052100 |
| FBgn0005626 | FBgn0011272 | FBgn0011661 | FBgn0041789 | FBgn0032731 | FBgn0040726 |
| FBgn0033869 | FBgn0003410 | FBgn0040985 | FBgn0003720 | FBgn0020912 | FBgn0004169 |
| FBgn0032378 | FBgn0039448 | FBgn0029943 | FBgn0037408 | FBgn0036382 | FBgn0052183 |
| FBgn0005633 | FBgn0013467 | FBgn0038931 | FBgn0031602 | FBgn0020618 | FBgn0020497 |
| FBgn0030052 | FBgn0028408 | FBgn0013799 | FBgn0020620 | FBgn0033317 | FBgn0032642 |
| FBgn0027492 | FBgn0035285 | FBgn0031646 | FBgn0046763 | FBgn0036761 | FBgn0002609 |
| FBgn0000625 | FBgn0031005 | FBgn0025800 | FBgn0003513 | FBgn0003975 | FBgn0031257 |
| FBgn0051145 | FBgn0031150 | FBgn0000395 | FBgn0004777 | FBgn0033931 | FBgn0000253 |
| FBgn0051163 | FBgn0002561 | FBgn0015320 | FBgn0000037 | FBgn0004551 | FBgn0001123 |
| FBgn0028969 | FBgn0003997 | FBgn0036134 | FBgn0026086 | FBgn0022268 | FBgn0033783 |
| FBgn0004646 | FBgn0032428 | FBgn0025743 | FBgn0038592 | FBgn0010105 | FBgn0004242 |
| FBgn0039359 | FBgn0035186 | FBgn0029764 | FBgn0030786 | FBgn0039831 | FBgn0053126 |
| FBgn0052105 | FBgn0002772 | FBgn0031836 | FBgn0034903 | FBgn0000024 | FBgn0039830 |
| FBgn0053094 | FBgn0000166 | FBgn0038145 | FBgn0000152 | FBgn0028563 | FBgn0000490 |
| FBgn0037721 | FBgn0029092 | FBgn0020309 | FBgn0015561 | FBgn0001994 | FBgn0052299 |
| FBgn0052698 | FBgn0051869 | FBgn0004436 | FBgn0011745 | FBgn0037551 | FBgn0033699 |
| FBgn0040079 | FBgn0016926 | FBgn0030364 | FBgn0031950 | FBgn0045064 | FBgn0039705 |
| FBgn0051361 | FBgn0002922 | FBgn0037972 | FBgn0033912 | FBgn0036341 | FBgn0033915 |
| FBgn0032856 | FBgn0031603 | FBgn0030680 | FBgn0003744 | FBgn0038118 | FBgn0030797 |
| FBgn0034504 | FBgn0029737 | FBgn0011297 | FBgn0026438 | FBgn0051100 | FBgn0019936 |
| FBgn0000258 | FBgn0043900 | FBgn0034539 | FBgn0040827 | FBgn0030672 | FBgn0017549 |
| FBgn0038658 | FBgn0004882 | FBgn0027581 | FBgn0032840 | FBgn0004638 | FBgn0000036 |
| FBgn0010516 | FBgn0039523 | FBgn0037351 | FBgn0011277 | FBgn0035429 | FBgn0035436 |
| FBgn0052372 | FBgn0016078 | FBgn0034650 | FBgn0039678 | FBgn0033961 | FBgn0031186 |
| FBgn0030976 | FBgn0032719 | FBgn0036391 | FBgn0003984 | FBgn0036032 | FBgn0014010 |
| FBgn0010114 | FBgn0024188 | FBgn0031850 | FBgn0002773 | FBgn0037424 | FBgn0039154 |
| FBgn0031826 | FBgn0011640 | FBgn0003612 | FBgn0014454 | FBgn0035060 | FBgn0043070 |
| FBgn0034013 | FBgn0017397 | FBgn0036801 | FBgn0026316 | FBgn0037698 | FBgn0035142 |
| FBgn0051140 | FBgn0003944 | FBgn0033971 | FBgn0041092 | FBgn0035016 | FBgn0052103 |
| FBgn0034674 | FBgn0039595 | FBgn0008646 | FBgn0004666 | FBgn0023170 | FBgn0036661 |
| FBgn0025879 | FBgn0025391 | FBgn0037926 | FBgn0022238 | FBgn0015721 | FBgn0037429 |
| FBgn0051291 | FBgn0004395 | FBgn0035600 | FBgn0030603 | FBgn0040600 | FBgn0052056 |
| FBgn0034889 | FBgn0034743 | FBgn0020235 | FBgn0026389 | FBgn0030038 | FBgn0020767 |
| FBgn0001138 | FBgn0035945 | FBgn0027556 | FBgn0019948 | FBgn0000575 | FBgn0010424 |
| FBgn0015806 | FBgn0037430 | FBgn0000633 | FBgn0005659 | FBgn0002629 | FBgn0039272 |
| FBgn0030529 | FBgn0038498 | FBgn0038389 | FBgn0028734 | FBgn0034644 | FBgn0051337 |
| FBgn0032083 | FBgn0003274 | FBgn0033129 | FBgn0003204 | FBgn0034602 | FBgn0031971 |
| FBgn0039151 | FBgn0026084 | FBgn0040376 | FBgn0039454 | FBgn0032666 | FBgn0032261 |
| FBgn0003319 | FBgn0035675 | FBgn0034645 | FBgn0038282 | FBgn0004841 | FBgn0036380 |
| FBgn0033726 | FBgn0017551 | FBgn0000171 | FBgn0035816 | FBgn0001197 | FBgn0000409 |
| FBgn0050271 | FBgn0029508 | FBgn0039844 | FBgn0051708 | FBgn0011259 | FBgn0004514 |
| FBgn0004908 | FBgn0034585 | FBgn0027932 | FBgn0016687 | FBgn0020496 | FBgn0020372 |
| FBgn0034570 | FBgn0038881 | FBgn0033551 | FBgn0033728 | FBgn0035954 | FBgn0037421 |
| FBgn0025463 | FBgn0034946 | FBgn0023535 | FBgn0037445 | FBgn0036967 | FBgn0045759 |
| FBgn0003267 | FBgn0036257 | FBgn0026597 | FBgn0032633 | FBgn0039584 | FBgn0011701 |
| FBgn0020445 | FBgn0013750 | FBgn0003475 | FBgn0029894 | FBgn0038043 | FBgn0027590 |
| FBgn0061198 | FBgn0000256 | FBgn0031090 | FBgn0036583 | FBgn0029152 | |
| FBgn0028420 | FBgn0026753 | FBgn0023528 | FBgn0050118 | FBgn0030766 | |
| FBgn0004868 | FBgn0037374 | FBgn0037262 | FBgn0030790 | FBgn0035517 | |
