# Supplementary figures and images for: Strong Purifying Selection at Synonymous Sites in D. melanogaster
Source: PLoS Genet. 2013 May 30;9(5):e1003527. doi: 10.1371/journal.pgen.1003527 (PMC3667748; doi:10.1371/journal.pgen.1003527)

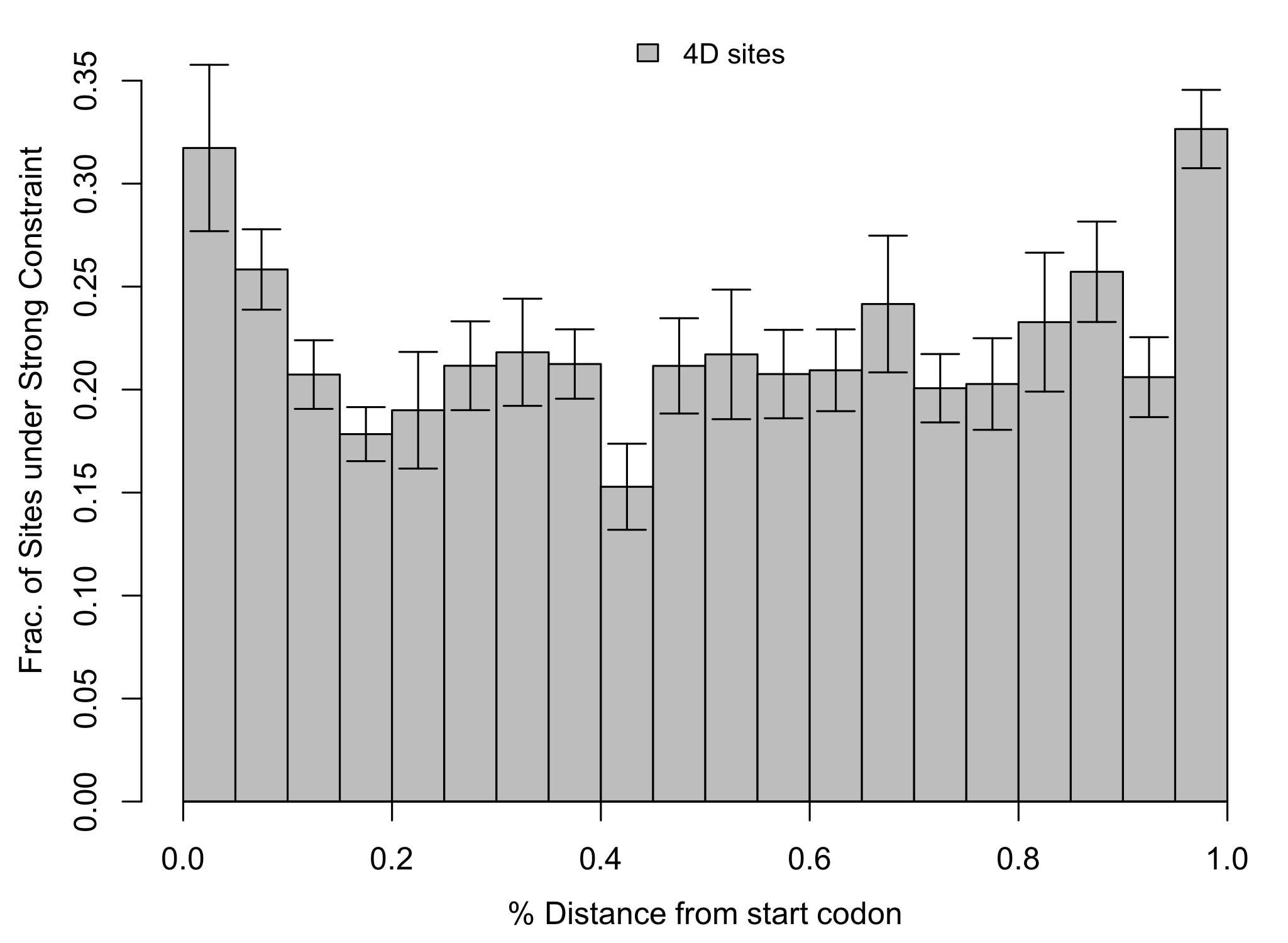

Supplement: Figure S1 — Spatial distribution of strong constraint within coding sequences. 4D sites were binned by their distance to the translation start site in the longest transcript for each gene. Each bin represents 5% of transcript length to control for different transcript lengths. 10 bootstraps to determine the fraction of sites under constraint were done within each bin. Error bars represent the s.e. of the estimates. (TIF) [file pgen.1003527.s002.tif]

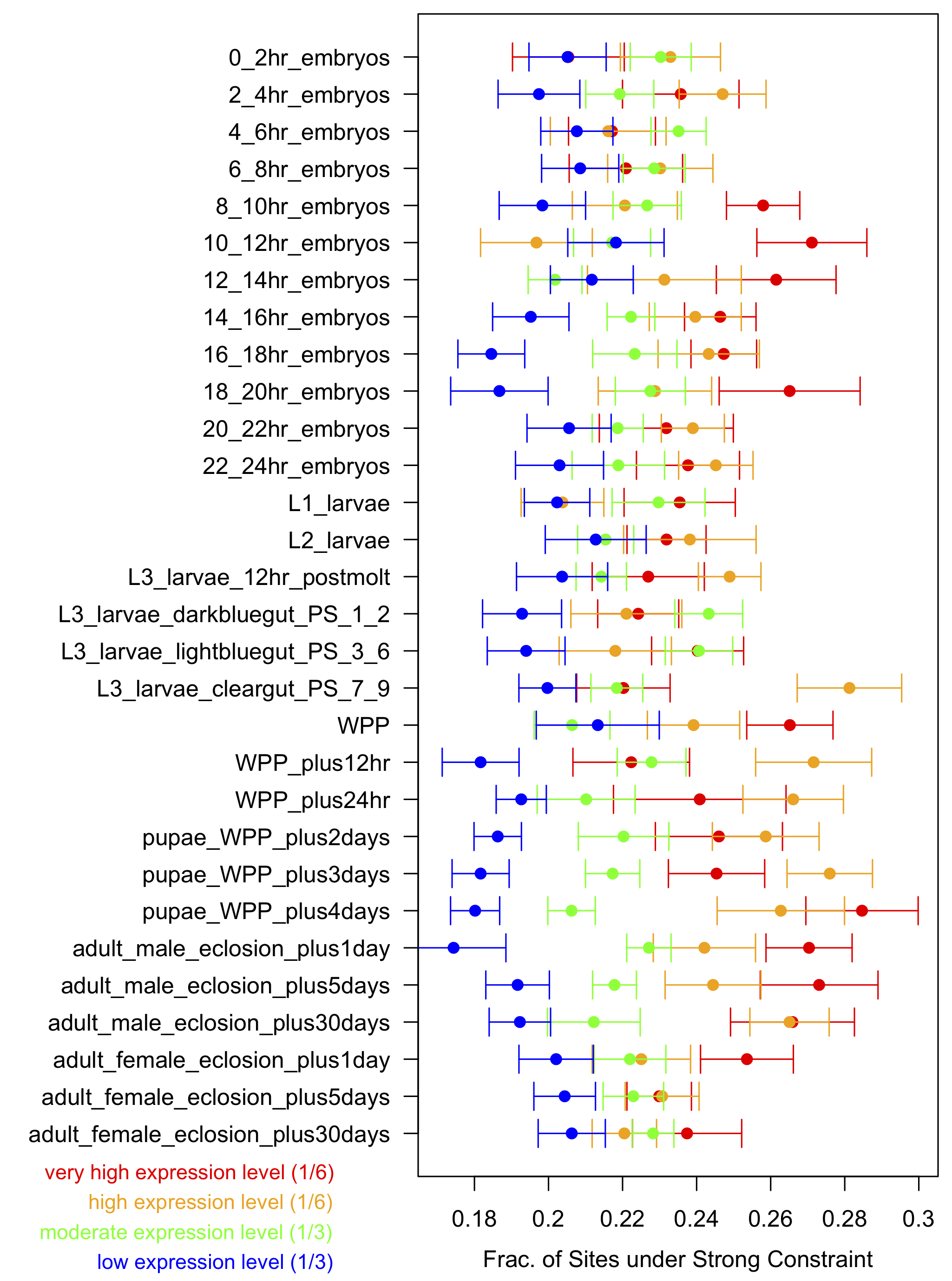

Supplement: Figure S2 — Strong constraint versus gene expression across development. Genes are grouped and analyzed as in Figure 5. Here, the Figure 5 “high expression level” gene set has been halved, creating a “very high expression level” and “high expression level” group, each containing one-sixth of all genes. Error bars represent the s.e. of the estimates. (TIF) [file pgen.1003527.s003.tif]
