# Supplementary material for: Strong Purifying Selection at Synonymous Sites in D. melanogaster
Source: PLoS Genet. 2013 May 30;9(5):e1003527. doi: 10.1371/journal.pgen.1003527 (PMC3667748; doi:10.1371/journal.pgen.1003527)
Supplement: Table S1 — Conservation versus constraint at 4D sites in conserved amino acids. The proportion of constrained 4D sites in each substitution class using fast-evolving 4D sites as the “neutral” reference and the relationship of those results to the proportion of constrained 4D sites in each substitution class using short introns as the reference. (DOC) [file pgen.1003527.s004.doc]

**Table S1. Conservation versus constraint at 4D sites in conserved amino acids.**

| **Rate Class^a^** | **Fraction of Sites^b^** | **Fraction of Sites^c^** | **Fraction of Sites^d^** |
| --- | --- | --- | --- |
| b_1_ = 0 | 27.1% (+/- 1.6%) | 13.7% (+/- 3.3%) | 40.8% (+/- 1.9%) |
| 0 < b_2_ ≤ 1.4 | 21.8% (+/- 2.3%) | 9.2% (+/- 3.4%) | 30.3% (+/- 3.0%) |
| 1.4 < b_3_ ≤ 1.92 | 18.1% (+/- 2.6%) | 9.0% (+/- 3.6%) | 29.5% (+/- 2.3%) |
| 1.92 < b_4_ ≤ 3.10 | 17.2% (+/- 2.6%) | 4.7% (+/- 3.1%) | 23.4% (+/- 2.7%) |
| 3.10 < b_5_ ≤ 4.40 | 10.7% (+/- 2.5%) | 10.3% (+/- 2.5%) | 18.0% (+/- 1.6%) |
| 4.40 < b_6_ ≤ 6.20 | 11.3% (+/- 2.4%) | 8.8% (+/- 2.5%) | 17.6% (+/- 2.0%) |
| 6.20 < b_7_ < 9.30 | 3.6% (+/- 2.4%) | 10.8% (+/- 2.4%) | 13.8% (+/- 2.1%) |
| b_8_ ≥ 9.30 | 0.7% (+/- 2.4%) | 6.5% (+/- 3.2%) | 7.1% (+/- 3.0%) |

^a^substitution rate classes of 4D sites in otherwise conserved amino acids; ^b^mean fraction of sites missing polymorphism over 10 bootstrap runs in 4D sites of each rate-class using fast-evolving 4D sites as reference (+/- s.e.); ^c^mean fraction of sites missing polymorphism relative to nearby short intron sites in those fast-evolving 4D sites used as the neutral reference for each rate-class; ^d^mean fraction of sites missing polymorphism in each rate class (short introns as neutral reference, same as reported in Figure 2).
