# Supplementary material for: Strong Purifying Selection at Synonymous Sites in D. melanogaster
Source: PLoS Genet. 2013 May 30;9(5):e1003527. doi: 10.1371/journal.pgen.1003527 (PMC3667748; doi:10.1371/journal.pgen.1003527)
Supplement: Table S2 — Estimated proportion of slow-evolving 4D sites and 4Nes for each selection class. Maximum likelihood results using fast-evolving 4D sites as the “neutral” reference. (DOC) [file pgen.1003527.s005.doc]

**Table S2. Estimated proportion of slow-evolving 4D sites and *4N_e_s* for each selection class.**

| **Selection Category**^a^ | **Fraction of Sites**^b^ | **Strength**^c^ |
| --- | --- | --- |
| Neutral | 84.2% (+/- 0.7%) | 0 |
| Weak Constraint | 0 | N/A |
| Strong Constraint | 15.8% (+/- 0.7%) | -307 (+/- 105) |

^a^selection categories are defined as follows => Neutral: 4N_e_s = 0, Weak Constraint: |4N_e_s| < 5, and Strong Constraint: |4N_e_s| > 100 (defining Strong Constraint: |4N_e_s| > 5 gives exactly the same MLE for the fraction/strength of the strong category); ^b^mean of the MLEs for the fraction of slow-evolving 4D sites in each category over the ten bootstrap runs (+/- s.e.); ^c^mean of the MLEs for the strength of strong selection over the ten bootstrap runs (+/- s.e.); 4N_e_μ (θ) = 0.0124
