# Supplementary material for: Strong Purifying Selection at Synonymous Sites in D. melanogaster
Source: PLoS Genet. 2013 May 30;9(5):e1003527. doi: 10.1371/journal.pgen.1003527 (PMC3667748; doi:10.1371/journal.pgen.1003527)
Supplement: Table S3 — Gene ontology clusters. Table of the full information for the top 13 GO clusters as reported by DAVID 6.7 from the 812 genes most enriched for strong constraint at 4D sites [86], [87]. (DOC) [file pgen.1003527.s006.doc]

**Table S3. Gene ontology clusters.**

| **Annotation Cluster 1** | **Enrichment Score: 9.691589451545648** | | | |  |  |  |  |  |  |
| --- | --- | --- | --- | --- | --- | --- | --- | --- | --- | --- |
| Category | Term | Count | | % | PValue | List Total | Pop Hits | Pop Total | Fold Enrichment | FDR |
| GOTERM_MF_FAT | GO:0030528~transcription regulator activity | 103 | | 12.68472906 | 2.52E-11 | 536 | 323 | 3078 | 1.831211589 | 3.74E-08 |
| GOTERM_BP_FAT | GO:0006355~regulation of transcription, DNA-dependent | 90 | | 11.08374384 | 3.88E-11 | 511 | 260 | 2817 | 1.908249285 | 6.67E-08 |
| GOTERM_BP_FAT | GO:0051252~regulation of RNA metabolic process | 95 | | 11.69950739 | 6.10E-10 | 511 | 293 | 2817 | 1.787400733 | 1.05E-06 |
| GOTERM_BP_FAT | GO:0045449~regulation of transcription | 105 | | 12.93103448 | 2.87E-09 | 511 | 344 | 2817 | 1.682661676 | 4.95E-06 |
|  |  |  | |  |  |  |  |  |  |  |
| **Annotation Cluster 2** | **Enrichment Score: 9.281593760361073** | | | |  |  |  |  |  |  |
| Category | Term | Count | | % | PValue | List Total | Pop Hits | Pop Total | Fold Enrichment | FDR |
| GOTERM_BP_FAT | GO:0007444~imaginal disc development | 69 | | 8.497536946 | 7.45E-14 | 511 | 159 | 2817 | 2.392312521 | 1.28E-10 |
| GOTERM_BP_FAT | GO:0002165~instar larval or pupal development | 70 | | 8.620689655 | 2.65E-11 | 511 | 180 | 2817 | 2.143835616 | 4.56E-08 |
| GOTERM_BP_FAT | GO:0048563~post-embryonic organ morphogenesis | 52 | | 6.403940887 | 5.66E-11 | 511 | 117 | 2817 | 2.450097847 | 9.75E-08 |
| GOTERM_BP_FAT | GO:0007560~imaginal disc morphogenesis | 52 | | 6.403940887 | 5.66E-11 | 511 | 117 | 2817 | 2.450097847 | 9.75E-08 |
| GOTERM_BP_FAT | GO:0009791~post-embryonic development | 70 | | 8.620689655 | 6.47E-11 | 511 | 183 | 2817 | 2.10869077 | 1.11E-07 |
| GOTERM_BP_FAT | GO:0048569~post-embryonic organ development | 52 | | 6.403940887 | 1.76E-10 | 511 | 120 | 2817 | 2.388845401 | 3.04E-07 |
| GOTERM_BP_FAT | GO:0007552~metamorphosis | 61 | | 7.512315271 | 3.88E-10 | 511 | 155 | 2817 | 2.169522126 | 6.67E-07 |
| GOTERM_BP_FAT | GO:0048737~imaginal disc-derived appendage development | 44 | | 5.418719212 | 7.13E-10 | 511 | 96 | 2817 | 2.526663405 | 1.23E-06 |
| GOTERM_BP_FAT | GO:0035114~imaginal disc-derived appendage morphogenesis | 44 | | 5.418719212 | 7.13E-10 | 511 | 96 | 2817 | 2.526663405 | 1.23E-06 |
| GOTERM_BP_FAT | GO:0009886~post-embryonic morphogenesis | 60 | | 7.389162562 | 8.93E-10 | 511 | 154 | 2817 | 2.147813048 | 1.54E-06 |
| GOTERM_BP_FAT | GO:0035107~appendage morphogenesis | 44 | | 5.418719212 | 1.07E-09 | 511 | 97 | 2817 | 2.500615329 | 1.84E-06 |
| GOTERM_BP_FAT | GO:0048736~appendage development | 44 | | 5.418719212 | 1.07E-09 | 511 | 97 | 2817 | 2.500615329 | 1.84E-06 |
| GOTERM_BP_FAT | GO:0048707~instar larval or pupal morphogenesis | 58 | | 7.142857143 | 1.90E-09 | 511 | 149 | 2817 | 2.145891068 | 3.27E-06 |
| GOTERM_BP_FAT | GO:0035120~post-embryonic appendage morphogenesis | 40 | | 4.926108374 | 1.00E-08 | 511 | 89 | 2817 | 2.477627037 | 1.72E-05 |
| GOTERM_BP_FAT | GO:0035220~wing disc development | 44 | | 5.418719212 | 1.45E-08 | 511 | 104 | 2817 | 2.332304682 | 2.50E-05 |
| GOTERM_BP_FAT | GO:0007476~imaginal disc-derived wing morphogenesis | 37 | | 4.556650246 | 5.29E-08 | 511 | 83 | 2817 | 2.45747766 | 9.11E-05 |
| GOTERM_BP_FAT | GO:0007472~wing disc morphogenesis | 37 | | 4.556650246 | 7.72E-08 | 511 | 84 | 2817 | 2.428221974 | 1.33E-04 |
|  |  |  | |  |  |  |  |  |  |  |
| **Annotation Cluster 3** | **Enrichment Score: 7.573942674767135** | | | |  |  |  |  |  |  |
| Category | Term | Count | % | | PValue | List Total | Pop Hits | Pop Total | Fold Enrichment | FDR |
| INTERPRO | IPR017970:Homeobox, conserved site | 26 | 3.201970443 | | 6.85E-09 | 691 | 47 | 4190 | 3.354373865 | 1.08E-05 |
| INTERPRO | IPR001356:Homeobox | 26 | 3.201970443 | | 6.85E-09 | 691 | 47 | 4190 | 3.354373865 | 1.08E-05 |
| SP_PIR_KEYWORDS | Homeobox | 26 | 3.201970443 | | 1.23E-08 | 786 | 48 | 4770 | 3.28721374 | 1.63E-05 |
| INTERPRO | IPR012287:Homeodomain-related | 25 | 3.078817734 | | 6.70E-08 | 691 | 48 | 4190 | 3.158164496 | 1.05E-04 |
| SMART | SM00389:HOX | 26 | 3.201970443 | | 3.48E-07 | 377 | 47 | 1869 | 2.742479824 | 4.34E-04 |
|  |  |  |  | |  |  |  |  |  |  |
| **Annotation Cluster 4** | **Enrichment Score: 7.4921762056957855** | | | | |  |  |  |  |  |
| Category | Term | Count | % | | PValue | List Total | Pop Hits | Pop Total | Fold Enrichment | FDR |
| GOTERM_BP_FAT | GO:0048592~eye morphogenesis | 41 | 5.049261084 | | 5.74E-09 | 511 | 91 | 2817 | 2.483753038 | 9.87E-06 |
| GOTERM_BP_FAT | GO:0007423~sensory organ development | 58 | 7.142857143 | | 8.07E-09 | 511 | 154 | 2817 | 2.07621928 | 1.39E-05 |
| GOTERM_BP_FAT | GO:0001745~compound eye morphogenesis | 37 | 4.556650246 | | 5.29E-08 | 511 | 83 | 2817 | 2.45747766 | 9.11E-05 |
| GOTERM_BP_FAT | GO:0001654~eye development | 47 | 5.78817734 | | 7.36E-08 | 511 | 120 | 2817 | 2.159148728 | 1.27E-04 |
| GOTERM_BP_FAT | GO:0048749~compound eye development | 44 | 5.418719212 | | 1.92E-07 | 511 | 112 | 2817 | 2.16571149 | 3.30E-04 |
|  |  |  |  | |  |  |  |  |  |  |
| **Annotation Cluster 5** | **Enrichment Score: 6.17076557737113** | | | |  |  |  |  |  |  |
| Category | Term | Count | % | | PValue | List Total | Pop Hits | Pop Total | Fold Enrichment | FDR |
| GOTERM_BP_FAT | GO:0001751~compound eye photoreceptor cell differentiation | 27 | 3.325123153 | | 5.25E-07 | 511 | 55 | 2817 | 2.70624444 | 9.05E-04 |
| GOTERM_BP_FAT | GO:0046530~photoreceptor cell differentiation | 28 | 3.448275862 | | 7.16E-07 | 511 | 59 | 2817 | 2.616206176 | 0.001232255 |
| GOTERM_BP_FAT | GO:0001754~eye photoreceptor cell differentiation | 27 | 3.325123153 | | 8.17E-07 | 511 | 56 | 2817 | 2.657918647 | 0.001406528 |
|  |  |  |  | |  |  |  |  |  |  |
| **Annotation Cluster 6** | **Enrichment Score: 6.070992847167416** | | | |  |  |  |  |  |  |
| Category | Term | Count | % | | PValue | List Total | Pop Hits | Pop Total | Fold Enrichment | FDR |
| GOTERM_BP_FAT | GO:0048729~tissue morphogenesis | 40 | 4.926108374 | | 1.75E-07 | 511 | 97 | 2817 | 2.273286662 | 3.02E-04 |
| GOTERM_BP_FAT | GO:0060429~epithelium development | 37 | 4.556650246 | | 8.56E-07 | 511 | 91 | 2817 | 2.241435668 | 0.001473044 |
| GOTERM_BP_FAT | GO:0002009~morphogenesis of an epithelium | 34 | 4.187192118 | | 4.08E-06 | 511 | 85 | 2817 | 2.205088063 | 0.007028429 |
|  |  |  |  | |  |  |  |  |  |  |
| **Annotation Cluster 7** | **Enrichment Score: 5.950492315603639** | | | |  |  |  |  |  |  |
| Category | Term | Count | % | | PValue | List Total | Pop Hits | Pop Total | Fold Enrichment | FDR |
| SP_PIR_KEYWORDS | transcription regulation | 63 | 7.75862069 | | 7.48E-08 | 786 | 197 | 4770 | 1.940752509 | 9.92E-05 |
| SP_PIR_KEYWORDS | Transcription | 63 | 7.75862069 | | 2.07E-07 | 786 | 202 | 4770 | 1.892714081 | 2.75E-04 |
| GOTERM_BP_FAT | GO:0006350~transcription | 66 | 8.128078818 | | 9.07E-05 | 511 | 234 | 2817 | 1.554869788 | 0.156037459 |
|  |  |  |  | |  |  |  |  |  |  |
| **Annotation Cluster 8** | **Enrichment Score: 5.930318493377584** | | | |  |  |  |  |  |  |
| Category | Term | Count | % | | PValue | List Total | Pop Hits | Pop Total | Fold Enrichment | FDR |
| INTERPRO | IPR003599:Immunoglobulin subtype | 19 | 2.339901478 | | 7.83E-08 | 691 | 30 | 4190 | 3.840328027 | 1.23E-04 |
| SMART | SM00409:IG | 19 | 2.339901478 | | 1.60E-06 | 377 | 30 | 1869 | 3.139787798 | 0.001993932 |
| INTERPRO | IPR007110:Immunoglobulin-like | 21 | 2.586206897 | | 1.29E-05 | 691 | 46 | 4190 | 2.768199836 | 0.020301505 |
|  |  |  |  | |  |  |  |  |  |  |
| **Annotation Cluster 9** | **Enrichment Score: 5.36295761301755** | | | |  |  |  |  |  |  |
| Category | Term | Count | % | | PValue | List Total | Pop Hits | Pop Total | Fold Enrichment | FDR |
| KEGG_PATHWAY | dme03010:Ribosome | 26 | 3.201970443 | | 1.36E-12 | 161 | 36 | 937 | 4.203243616 | 1.37E-09 |
| GOTERM_CC_FAT | GO:0022626~cytosolic ribosome | 26 | 3.201970443 | | 8.76E-12 | 336 | 36 | 1869 | 4.017361111 | 1.21E-08 |
| GOTERM_CC_FAT | GO:0044445~cytosolic part | 27 | 3.325123153 | | 8.73E-07 | 336 | 57 | 1869 | 2.634868421 | 0.001205052 |
| SP_PIR_KEYWORDS | ribonucleoprotein | 28 | 3.448275862 | | 1.72E-06 | 786 | 66 | 4770 | 2.574600972 | 0.002282836 |
| SP_PIR_KEYWORDS | ribosomal protein | 31 | 3.81773399 | | 4.51E-05 | 786 | 89 | 4770 | 2.113817652 | 0.059807775 |
| GOTERM_CC_FAT | GO:0005840~ribosome | 34 | 4.187192118 | | 8.61E-05 | 336 | 98 | 1869 | 1.929846939 | 0.118777504 |
| GOTERM_CC_FAT | GO:0033279~ribosomal subunit | 32 | 3.9408867 | | 8.78E-05 | 336 | 90 | 1869 | 1.977777778 | 0.121142593 |
| GOTERM_MF_FAT | GO:0003735~structural constituent of ribosome | 32 | 3.9408867 | | 1.15E-04 | 536 | 93 | 3078 | 1.975926818 | 0.170384523 |
| GOTERM_BP_FAT | GO:0006412~translation | 39 | 4.802955665 | | 0.050115863 | 511 | 163 | 2817 | 1.318994393 | 58.72942407 |
| GOTERM_CC_FAT | GO:0030529~ribonucleoprotein complex | 41 | 5.049261084 | | 0.066869989 | 336 | 179 | 1869 | 1.274092179 | 61.53930522 |
|  |  |  |  | |  |  |  |  |  |  |
| **Annotation Cluster 10** | **Enrichment Score: 4.591159300959751** | | | |  |  |  |  |  |  |
| Category | Term | Count | % | | PValue | List Total | Pop Hits | Pop Total | Fold Enrichment | FDR |
| GOTERM_BP_FAT | GO:0007267~cell-cell signaling | 35 | 4.310344828 | | 1.37E-05 | 511 | 93 | 2817 | 2.074679629 | 0.023625556 |
| GOTERM_BP_FAT | GO:0007268~synaptic transmission | 32 | 3.9408867 | | 2.68E-05 | 511 | 84 | 2817 | 2.100083869 | 0.046138564 |
| GOTERM_BP_FAT | GO:0019226~transmission of nerve impulse | 32 | 3.9408867 | | 4.58E-05 | 511 | 86 | 2817 | 2.051244709 | 0.078769078 |
|  |  |  |  | |  |  |  |  |  |  |
| **Annotation Cluster 11** | **Enrichment Score: 4.410252952822596** | | | |  |  |  |  |  |  |
| Category | Term | Count | % | | PValue | List Total | Pop Hits | Pop Total | Fold Enrichment | FDR |
| GOTERM_BP_FAT | GO:0046552~photoreceptor cell fate commitment | 16 | 1.97044335 | | 2.22E-05 | 511 | 28 | 2817 | 3.150125804 | 0.038285718 |
| GOTERM_BP_FAT | GO:0048663~neuron fate commitment | 16 | 1.97044335 | | 2.22E-05 | 511 | 28 | 2817 | 3.150125804 | 0.038285718 |
| GOTERM_BP_FAT | GO:0001752~compound eye photoreceptor fate commitment | 15 | 1.84729064 | | 6.80E-05 | 511 | 27 | 2817 | 3.062622309 | 0.116912828 |
| GOTERM_BP_FAT | GO:0042706~eye photoreceptor cell fate commitment | 15 | 1.84729064 | | 6.80E-05 | 511 | 27 | 2817 | 3.062622309 | 0.116912828 |
|  |  |  |  | |  |  |  |  |  |  |
| **Annotation Cluster 12** | **Enrichment Score: 4.327193195181524** | | | |  |  |  |  |  |  |
| Category | Term | Count | % | | PValue | List Total | Pop Hits | Pop Total | Fold Enrichment | FDR |
| GOTERM_BP_FAT | GO:0048609~reproductive process in a multicellular organism | 69 | 8.497536946 | | 5.22E-06 | 511 | 229 | 2817 | 1.661037951 | 0.00898789 |
| GOTERM_BP_FAT | GO:0032504~multicellular organism reproduction | 69 | 8.497536946 | | 5.22E-06 | 511 | 229 | 2817 | 1.661037951 | 0.00898789 |
| GOTERM_BP_FAT | GO:0007276~gamete generation | 63 | 7.75862069 | | 3.94E-05 | 511 | 215 | 2817 | 1.615355209 | 0.067752345 |
| GOTERM_BP_FAT | GO:0019953~sexual reproduction | 63 | 7.75862069 | | 5.37E-05 | 511 | 217 | 2817 | 1.600467142 | 0.092370275 |
| GOTERM_BP_FAT | GO:0048610~reproductive cellular process | 51 | 6.280788177 | | 8.20E-05 | 511 | 167 | 2817 | 1.683525317 | 0.140974756 |
| GOTERM_BP_FAT | GO:0007292~female gamete generation | 50 | 6.157635468 | | 3.19E-04 | 511 | 171 | 2817 | 1.611906479 | 0.547637831 |
| GOTERM_BP_FAT | GO:0048477~oogenesis | 49 | 6.034482759 | | 3.40E-04 | 511 | 167 | 2817 | 1.617504717 | 0.583843907 |
|  |  |  |  | |  |  |  |  |  |  |
| **Annotation Cluster 13** | **Enrichment Score: 3.5098655719773366** | | | | |  |  |  |  |  |
| Category | Term | Count | % | | PValue | List Total | Pop Hits | Pop Total | Fold Enrichment | FDR |
| GOTERM_BP_FAT | GO:0048666~neuron development | 48 | 5.911330049 | | 2.12E-05 | 511 | 147 | 2817 | 1.800071888 | 0.036521547 |
| GOTERM_BP_FAT | GO:0000904~cell morphogenesis involved in differentiation | 43 | 5.295566502 | | 4.52E-05 | 511 | 130 | 2817 | 1.823438206 | 0.077726513 |
| GOTERM_BP_FAT | GO:0048667~cell morphogenesis involved in neuron differentiation | 40 | 4.926108374 | | 1.35E-04 | 511 | 123 | 2817 | 1.792754522 | 0.23201353 |
| GOTERM_BP_FAT | GO:0048812~neuron projection morphogenesis | 39 | 4.802955665 | | 2.06E-04 | 511 | 121 | 2817 | 1.776827158 | 0.354155351 |
| GOTERM_BP_FAT | GO:0031175~neuron projection development | 39 | 4.802955665 | | 2.49E-04 | 511 | 122 | 2817 | 1.762263001 | 0.428191192 |
| GOTERM_BP_FAT | GO:0048858~cell projection morphogenesis | 39 | 4.802955665 | | 0.001609619 | 511 | 133 | 2817 | 1.616511926 | 2.734806782 |
| GOTERM_BP_FAT | GO:0030030~cell projection organization | 42 | 5.172413793 | | 0.002346925 | 511 | 149 | 2817 | 1.553921118 | 3.963852685 |
| GOTERM_BP_FAT | GO:0032990~cell part morphogenesis | 39 | 4.802955665 | | 0.003321885 | 511 | 138 | 2817 | 1.557942653 | 5.566603734 |
