# Supplementary material for: Strong Purifying Selection at Synonymous Sites in D. melanogaster
Source: PLoS Genet. 2013 May 30;9(5):e1003527. doi: 10.1371/journal.pgen.1003527 (PMC3667748; doi:10.1371/journal.pgen.1003527)
Supplement: Text S1 — Demographic correction of SFS. Maximum likelihood results when correcting for demography and other non-mutation-selection balance forces in the SFS. (DOC) [file pgen.1003527.s007.doc]

**Text S1 – Demographic correction of SFS**

We used a frequency-dependent variable α to correct for demography, linked selection, weak selection affecting both test and reference sites, and our approximation to the true SFS (see Materials and Methods). The frequency correction adds 64 additional free variables over which the likelihood needs to be maximized (see Materials and Methods). Using fminsearch in Matlab as before to perform the maximum-likelihood (see Materials and Methods), we ran several different variable initializations of *f* (fraction of sites in each category), *γ* (strength of selection, *4N_e_s*, in each category), and *θ* (effective mutation rate, *4N_e_μ*) for each of the 10 bootstraps and then chose the local maxima with the lowest log likelihood out of the initializations. These reported values thus cannot be guaranteed to be the global maxima for each bootstrap.

*f* = (*f_neutral_*, *f_weak_* , *f_strong_*); *γ* = (*γ_weak_*, *γ_strong_*); *θ*

0) *f* = (0.7832, 0.0086, 0.2082); *γ* = (-0.7391, -531.2); *θ* = 0.0137

1) *f* = (0.7682, 0.0046, 0.2272); *γ* = (-3.8237, -497.0); *θ* = 0.0140

2) *f* = (0.7710, 0.0094, 0.2196); *γ* = (-3.6057, -332.4); *θ* = 0.0135

3) *f* = (0.7642, 0.0107, 0.2251); *γ* = (-4.6512, -324.5); *θ* = 0.0137

4) *f* = (0.7697, 0.0010, 0.2293); *γ* = (-0.0018, -295.1); *θ* = 0.0137

5) *f* = (0.7667, 0.0089, 0.2244); *γ* = (-0.1215, -336.1); *θ* = 0.0137

6) *f* = (0.7784, 0.0106, 0.2110); *γ* = (-4.6003, -351.5); *θ* = 0.0138

7) *f* = (0.7614, 0.0090, 0.2296); *γ* = (-0.0003, -310.8); *θ* = 0.0141

8) *f* = (0.7677, 0.0128, 0.2195); *γ* = (-4.0697, -507.4); *θ* = 0.0138

9) *f* = (0.7475, 0.0231, 0.2294); *γ* = (-0.0002, -215.2); *θ* = 0.0136

avg % of sites under strong selection: 22.23% +/- 0.767 (+/- s.e.)

avg strength of strong selection: -370.12 +/- 105 (+/- s.e.)

As compared to the estimates without demographic correction, the percent of sites under strong selection is roughly the same, but the strength of that selection is somewhat higher. The variance of that latter estimation is unsurprisingly larger given the increased number of variables. The estimated intensity of the strong constraint is however still greater than three times the standard error away from both -700, the calculable limit of our program, and weak selection (-5).
