# Supplementary material for: Strong Purifying Selection at Synonymous Sites in D. melanogaster
Source: PLoS Genet. 2013 May 30;9(5):e1003527. doi: 10.1371/journal.pgen.1003527 (PMC3667748; doi:10.1371/journal.pgen.1003527)
Supplement: Text S2 — Mutation versus strong constraint. Discusses extra controls for distinguishing the strong constraint from possible confounding mutational effects: S2.A – Tri-nucleotide bootstrap results, S2.B – Power to detect low mutation versus strong purifying selection on 4D sites, S2.C – Slow- versus fast-evolving 4D site bootstrap results. (DOC) [file pgen.1003527.s008.doc]

**Text S2 – Mutation versus strong constraint**

*S2.A – Tri-nucleotide bootstrap results*

To test if the lack of polymorphism at 4D sites relative to short introns could be explained by a lower context-dependent mutation rate at 4D sites, we ran bootstraps matching the 4D sites and their flanking nucleotides with nearby (again, < 1KB) triplets of short intron sites. We split the results up by whether the 4D sites were in conserved versus variable amino acids (conservation is across the 12 Drosophila species tree). In the 4D sites of otherwise conserved amino acids, 18.5% (+/- 1.5%) (+/- s.e.) of sites are missing polymorphism. For 4D sites in variable amino acids, the percentage of missing polymorphism drops to 16.6% (+/- 1.1%). So matching triplets to control for dinucleotide content would appear to explain some fraction of the overall signal of missing polymorphism. However, closer analysis reveals this to be an artifact of the type of 4D site capable of matching nearby short introns when controlling for neighbors. The GC content of all 4D sites falling in conserved and variable amino acids is 67.1% and 61.9% respectively, while the GC content of those 4D sites capable of matching neighbors with short introns is ~61.3% and ~55.8% respectively. The GC content of short introns is far lower than that of 4D sites and thus 4D sites with G or C as their major allele have more trouble finding nearby short intron G/C sites with the same flanking nucleotides. Thus the makeup of the 4D sites capable of being used in the in the tri-nucleotide bootstrap is skewed compared to the full sample. Just taking those 4D sites from the tri-nucleotide bootstrap and matching them instead to short intron sites with the same major allele, but not the same flanking nucleotides reveals a nearly identical amount of missing polymorphism to the above: 19.5% (+/- 0.9%) and 16.6% (+/- 0.9%) for 4D sites in conserved and variable amino acids respectively. The matched short intron sites now have a different neighborhood than the 4D sites, but the resulting drop in polymorphism is the same. In the triplet bootstrap, the sample of 4D sites is biased by the neighborhood control rather than the neighborhood controlling for a context-dependent mutation rate. As such, dinucleotide biases and consequent context-dependent mutational effects do not explain any appreciable amount of the drop in polymorphism in 4D sites relative to short introns.

*S2.B – Power to detect low mutation versus strong purifying selection on 4D sites*

We simulated three sample spectra matching the total number of sites and the depth of population sample to that observed in the data: a neutral reference SFS, a low-mutation SFS, and a strong constraint SFS (see Materials and Methods). We used the first as a reference against which to measure the apparent amount of selection acting on the latter two. For the neutral, but 22%-lower-mutation-rate SFS, we estimated the strength of effective selection to be *4N_e_s* ~ -700 on 22% of sites, the calculable limit of our program for Drosophila-like parameters and thus essentially infinitely strong purifying selection. In the third SFS, 22% of the sites evolved under a constraint of *4N_e_s* = -283 with the rest neutral. Re-estimating the intensity of the strong constraint category to be -283 provides a significantly better fit to the SFS than setting its value equal to -700 *a priori* (p-value: 0.0366, LRT ~ X^2^_1_). When the strong selection category is set *a priori* to be infinitely strong, the maximum-likelihood procedure compensates for the slight differences between the short intron and 4D site spectra with small amounts of weak selection. If we remove the weak selection category from the maximum-likelihood analysis, the above likelihood differential increases in favor of a finite strong selective force over mutational force explaining the SFS (p-value: 0.0137, LRT ~ X^2^_1_). So while weak selection can buoy the likelihood of a mutational force explaining the spectra, we have enough sites from across the genome and samples from within a population to distinguish between a SFS under a finite, strong selective force and a low-mutation SFS.

*S2.C – Slow- versus fast-evolving 4D site bootstrap results*

Using the same intervals as Figure 2, we can use the fastest evolving 4D sites in otherwise conserved amino acids, class b_8_ in Figure 2, as the neutral reference against which to measure the amount of missing polymorphism in 4D sites across the different substitution-rate classes (Table S1). We matched slow-evolving and fast-evolving 4D sites as before by major allele, controlling for distance (< 1KB). When matching fast-evolving 4D sites to each other in the bootstrap, we prohibited a 4D site from matching with itself. As shown in Figure 2, the fast-evolving 4D sites are not a perfect neutral reference – a few are themselves under strong constraint – so the percent of missing polymorphism in fast-evolving 4D sites used as the neutral reference for each rate-class was measured relative to short introns. Overall it is 7.1% (+/- 3.0%). Summing the two columns of missing polymorphism together in Table S1 (S1.b + S1.c) almost perfectly recapitulates the fraction of sites under strong constraint as measured in Figure 2 (S1.d).

Applying our SFS model (w/o frequency-dependent correction) to test all slow-evolving 4D sites (< 9.3) for selection, using fast-evolving 4D sites (>= 9.3) as neutral reference, yields the result in Table S2. Table S2 shows that our results are not dependent on using short introns as a neutral reference – that they are not due to any artifact of short introns. Remaining strong constraint on fast-evolving 4D sites results in a more conservative estimate of the fraction of sites under constraint when the fast-evolving sites are used as the “neutral” reference, but regardless there is both less polymorphism at slower-evolving 4D sites than at fast-evolving 4D sites and a signal of strong constraint in their relative site-frequency spectra. Further tests were done matching neighborhoods of slow and fast evolving 4D sites in the bootstrap and as in S2.A, no significant effects on the amount of missing polymorphism were found (not shown).
