# Supplementary material for: Strong Purifying Selection at Synonymous Sites in D. melanogaster
Source: PLoS Genet. 2013 May 30;9(5):e1003527. doi: 10.1371/journal.pgen.1003527 (PMC3667748; doi:10.1371/journal.pgen.1003527)
Supplement: Text S3 — Weak selection on bulk nucleosomes. Evidence suggests a selective force operating in regions bound by bulk nucleosomes, but that the force appears to be different from the strong purifying selection inferred in the main result of this paper. (DOC) [file pgen.1003527.s009.doc]

**Text S3 – Weak selection on bulk nucleosomes**

Bulk nucleosomes cover about 67% of our 4D sites. However, 55% of our short introns positions are also bound by nucleosomes. Comparing the level of polymorphism in nucleosomal to non-nucleosomal short intron positions, we see a 9.0% drop even before accounting for the GC differences between them. Further the average minor allele frequencies of SNPs in nucleosomal short intron positions is lower than those of SNPs in non-nucleosomal short intron positions – a small, but significant shift of 0.1353 vs. 0.1424 respectively, a difference outside of each estimate’s 95% confidence intervals as determined by 80 bootstraps – potentially indicative of weak selection. As such, while 24% of polymorphism may be missing in 4D sites bound nucleosomes relative to all short intron positions, the fraction of sites under strong constraint is still truly ~22%. Our previous SFS analysis showed no sign of weak selection on 4D sites, but bulk nucleosomes cover short introns to almost the same extent as 4D sites. The signal from any selective or mutational force caused by nucleosomes is washed out when comparing 4D sites with short introns. Thus the binding of bulk nucleosomes does not explain the signal of strong constraint in 4D sites when compared to short introns. We did not see a correlation of strong constraint with active, H2A.Z nucleosomal binding or PolII binding (not shown).
