# Supplementary material for: Strong Purifying Selection at Synonymous Sites in D. melanogaster
Source: PLoS Genet. 2013 May 30;9(5):e1003527. doi: 10.1371/journal.pgen.1003527 (PMC3667748; doi:10.1371/journal.pgen.1003527)
Supplement: Text S4 — Phylogenetic tree and parameters of 4D sites. The 12 Drosophila species tree and nucleotide substitution parameters inferred on 4D synonymous sites. (DOC) [file pgen.1003527.s010.doc]

**Text S4 – Phylogenetic tree and parameters of 4D sites**

Below is the ascertained tree for 4D sites in *D. melanogater*, representing the average rate of evolution along each branch for 4D sites in otherwise conserved amino acids:

(((((dm3:0.0570839230,(droSim1:0.0191559629,droSec1:0.0228909640):0.0245005060):0.0521408013,(droYak2:0.0880756403,droEre2:0.0765664754):0.0327248791):0.3298133685,droAna3:0.4828717943):0.1740811596,(dp4:0.0119588627,droPer1:0.0118166618):0.4002579069):0.1152779250,droWil1:0.6852845130,((droVir3:0.2436546904,droMoj3:0.3505049405):0.0822301249,droGri2:0.3355523333):0.2483077527);

dm3 – *D. melanogaster*, droSim1 – *D. simulans*, droSec1 – *D. sechellia,* droYak2 – *D. yakuba*, droEre2 – *D. erecta*, droAna3 – *D. ananassae*, dp4 – *D. pseudoobscura*, droPer1 – *D. persimilis*, droWil1 – *D. willistoni*, droVir3 – *D. virilis*, droMoj3 – *D. mojavensis*, droGri2 – *D. grimshawi*

transition/transversion rate ratio, k = 1.865

π_A_ = the probability of being in state A

π_A_ = 0.19985

π_C_ = 0.38418

π_G_ = 0.23557

π_T_ = 0.18040

GERP uses a different parameterization of the transition-transversion ratiofrom that used by PhyML. (citations) Using the HKY85 model (citation), PhyML’s k is a rate modifier for transitions (i.e. r_A->G_ = k*π_G_), while GERP’s R is the ratio in overall frequency of change (i.e. f_A->G_ = π_A_*r_A->G_ – the probability of being in A and going to G) between transitions and transversions (i.e. R = f_transiution_/f_transversion_).

The two parameterizations, R (GERP) and k (PhyML) are related as follows:

R = k*(π_A_*π_G_ + π_C_*π_T_)/(π_X_*π_Y_)

π_X_ = π_A_ + π_G_

π_Y_ = π_C_ + π_T_

 R = 0.8830
